# A Multiresolution PDE-Based Deformable Surface for Medical Imaging Applications

**DOI:** 10.1155/IJBI/2006/87419

**Published:** 2006-04-18

**Authors:** Ye Duan

**Affiliations:** Computer Graphics and Image Understanding Lab, Department of Computer Science, College of Engineering, University of Missouri-Columbia, Columbia, MO 65211-2060, USA

## Abstract

We recently developed a multiresolution PDE-based deformable surface whose deformation behavior is governed by partial differential equations (PDEs) such as the weighted minimal surface flow. Comparing with the level-set approach, our new model has better control of the mesh quality and model resolution, and is much simpler to implement since all the computations are local. The new deformable model is very useful for a variety of medical imaging applications including boundary reconstruction, surface visualization, data segmentation, and topology discovery. In this paper, we demonstrate both the accuracy and robustness of our model on areas such as medical image segmentation through a number of experiments on both real (MRI/CT) and synthetic volumetric datasets.


Hindawi Publishing Corporation
International Journal of Biomedical Imaging
Volume 2006, Article ID 87419, Pages 1–7
DOI 10.1155/IJBI/2006/87419

### A Multiresolution PDE-Based Deformable Surface for Medical Imaging Applications

**Ye Duan**

*Computer Graphics and Image Understanding Lab, Department of Computer Science, College of Engineering, University of Missouri-Columbia, Columbia, MO 65211-2060, USA*

Received 31 July 2005; Revised 19 December 2005; Accepted 20 December 2005

Recommended for Publication by Guowei Wei

We recently developed a multiresolution PDE-based deformable surface whose deformation behavior is governed by partial differential equations (PDEs) such as the weighted minimal surface flow. Comparing with the level-set approach, our new model has better control of the mesh quality and model resolution, and is much simpler to implement since all the computations are local. The new deformable model is very useful for a variety of medical imaging applications including boundary reconstruction, surface visualization, data segmentation, and topology discovery. In this paper, we demonstrate both the accuracy and robustness of our model on areas such as medical image segmentation through a number of experiments on both real (MRI/CT) and synthetic volumetric datasets.

Copyright © 2006 Ye Duan. This is an open access article distributed under the Creative Commons Attribution License, which permits unrestricted use, distribution, and reproduction in any medium, provided the original work is properly cited.

### 1. INTRODUCTION

Since the seminal work of the snake model proposed by Kass et al. [1] in 1988, deformable models have significantly gained popularity in medical imaging analysis and related fields. Conventional deformable models are essentially parametric models [2–4]. In general, they have concise representation and it is easy to incorporate user constraints in them. However, it is very difficult for parameterized deformable models to represent shapes of arbitrary topology. Recently, implicit snakes were proposed by Malladi et al. [5] and Caselles et al. [6] with the ability to handle topology changes. Their schemes are based on the modeling of propagating fronts which are the level set of certain scalar functions. Nonetheless, the topological flexibility is accompanied by a time-consuming integration method that must be applied to a higher-dimensional space, and the desirable shape must be explicitly evaluated using the marching-cube-like [3] techniques in a separate postprocessing stage.

In order to bridge the divide between explicit parametric models and implicit level-set models [7, 8], most recently, researchers have proposed a new kind of deformable model—topology-adaptive explicit deformable models such as the topology-adaptive snake proposed by McInerney and Terzopoulos [9, 10] and the discrete triangle model of Lachaud and Montanvert [11]. Following the similar research direction, we had also developed a new topology-adaptive explicit deformable model. Our new model is a multiresolution PDE-based deformable surface whose deformation behavior is governed by PDEs such as the weighted minimal surface flow. The new model can either grow from the inside or shrink from the outside. Furthermore, our new model supports different LODs through the use of both global subdivision and local/adaptive subdivision. Comparing with existing topology-adaptive explicit models [9–11], our new model has better control of the mesh quality and model resolution, and is much simpler to implement since all the computations are local. The new deformable model will be very useful for a variety of medical image applications including boundary reconstruction, surface visualization, data segmentation, and topology discovery. In this paper, we illustrate some of the experimental results of our new model on medical image segmentation for both real (MRI/CT) and synthetic volumetric datasets.

Since the proposed deformation framework is quite general, it can also be applied to other application domains that are outside the scope of biomedical imaging. For example, it can be applied for 3D shape reconstruction from 2D

multiple-view images and unorganized point clouds [12]. It can also be applied for interactive mesh editing, sketching, morphing, as well as shape warping [13].

### 2. ALGORITHM

The entire algorithmic pipeline of our new deformable model consists of the following major steps:

(1) model initialization;
(2) model growing;
(3) model relaxation;
(4) topology modification;
(5) model refinement.

### 2.1. Model initialization

The model can be initialized either interactively by the user as a small seed model that can grow from the inside, or automatically by the system as a bounding box that will shrink from the outside. Any closed polyhedron can be used as a seed model. If the model is initialized as a simple bounding box shrink-wrapping from the outside, several iterations of global refinement are usually needed to increase the model resolution so as to correctly capture the geometry and topology of the shape. In this paper, we use Loop's subdivision scheme [14] for the purpose of global refinement.

### 2.2. Model growing

After the model is initialized, the model will grow (or shrink) and deform. The deformation of the model is governed by a PDE called weighted minimal surface flow proposed by Caselles et al. [6]:

$$\frac{\partial S}{\partial t} = \left((\nu + H)g - \nabla g \cdot \overset{\rho}{N}\right)\overset{\rho}{N}, \qquad S(0) = S_0. \tag{1}$$

$S = S(t)$ is the 3D deformable surface, $t$ is the time parameter, and $S_0$ is the initial shape of the surface. $H$ is the mean curvature value of the surface, $\overset{\rho}{N}$ is the unit normal vector of the surface, and $\nu$ is a constant speed that will enable the convex initial shape to capture nonconvex, arbitrary complicated shapes. The nonzero constant velocity is also useful to avoid the model getting stuck into the local minimum during the evolution process. $g$ is the monotonic, nonincreasing, nonnegative weight function that enables the model to interact with the datasets, and will stop the deformation of the model when it reaches the object boundary. In this paper, $g$ is defined as the commonly used 3D edge detector:

$$g(S) = \frac{1}{1 + \left|\nabla\left(G_\sigma * I(S)\right)\right|^2}. \tag{2}$$

Here, $I$ is the volumetric density function, and $G_\sigma * I$ is the smoothed density function by convoluting with a Gaussian filter with variance $\sigma$. The surface evolution process is approximated using an explicit iterative equation:

$$S(p, t + \Delta t) = S(p, t) + F(p, t)\Delta t, \tag{3}$$

where $F(p, t)$ is the evolution speed of the surface at the current position $p$ and at the current time $t$, and is calculated by the right-hand side of (1). To calculate the mean curvature of the surface, we employ the discrete curvature estimator proposed by Desbrun et al. [15]:

$$H = \frac{1}{\sum_{j\in N_1(i)}\left(\cot\alpha_j + \cot\beta_j\right)} \times \sum_{j\in N_1(i)}\left(\cot\alpha_j + \cot\beta_j\right)\left(x_i - x_j\right). \tag{4}$$

Here $x_j$ is one of the vertices at the one-neighborhood of $x_i$. $\alpha_j$ and $\beta_j$ are the two angles opposite to the edge connecting the two vertices $x_i$ and $x_j$, and $H$ is the mean curvature vector at vertex $x_i$. In essence, (1) controls how each point in the deformable surface should move in order to minimize the weighted surface area. The detected object is then reconstructed by the steady-state solution of the equation $S_t = 0$ (i.e., the velocity is zero).

In order to control both the smoothness of the model and the size of each triangle during the model-deformation phase, we must allow the model to be able to dynamically change its degrees of freedom during the deformation process. This is achieved using the local subdivision. If the area of an active face (on the model) is larger than a certain user-defined threshold, then this face will be subdivided into four smaller triangles by splitting the middle positions of its three edges.

### 2.3. Model relaxation

To ensure that the numerical simulation of the deformation process proceeds smoothly, we must maintain the regularity of the mesh such that the mesh has a good node distribution, a proper node density, and a good aspect ratio of the triangles. This is achieved by the incorporation of the tangential Laplacian operator, and three mesh operations: edge split, edge collapse, and edge swap. Laplacian operator, in its simplest form, moves repeatedly each mesh vertex by a displacement equal to a positive scale factor times the average of the neighboring vertices. Consider a mesh vertex $P$ and its neighbors $Q_1, \Lambda, Q_n$, the Laplacian operator $U$ is

$$U(p) = \frac{1}{n}\sum_{i=1}^{n} Q_i - P. \tag{5}$$

The tangential Laplacian operator is used to maintain a good node distribution and is defined as

$$T(p) = C[U - (U \bullet n)n], \tag{6}$$

where $n$ is the mesh normal at vertex $P$ and $C$ is a positive constant.

Edge split and edge collapse are used to keep an appropriate node density. An edge split is triggered if the edge length is bigger than the maximum edge length threshold. Similarly, an edge will be collapsed if its length is smaller than the minimum edge length threshold. Edge swapping is used to ensure a good aspect ratio of the triangles. As suggested by Kobbelt et al. [16], this can be achieved by forcing the average valence to be as close to 6 as possible. An edge is swapped if and only if the quantity $\sum_{p\in\Delta}(\text{valence}(p)-6)^2$ is minimized after the swapping.

### 2.4. Topology modification

In order to recover a shape of arbitrary, unknown topology, the model must be able to modify its topology properly whenever necessary. In general, there are two kinds of topology operations: (1) topology merging, and (2) topology splitting. We will explain these two operations in the following two subsections, respectively.

#### 2.4.1. Topology merging

We propose a novel method called "*lazy merging*" to handle topology merging. The basic idea is that whenever two nonneighboring vertices are too close to each other, they will be deactivated (i.e., not allowed to move). Topology merging will happen only after the deformation of the model stops and all the vertices become nonactive. There are three steps in the topology merging operation: (1) *collision detection*, (2) *merging-vertices clustering*, and (3) *multiple-contours stitching*.

*Collision detection*

Collision detection is done hierarchically in two different levels: coarser-level and finer-level. Coarser-level collision detection is mainly for the purpose of collision exclusion. For each active vertex $V$, we will calculate its distance to all other nonneighboring active vertices. Vertices whose distance to the current vertex $V$ is bigger enough so that no collision will happen between them will be excluded from collision detection in the next level. Otherwise, they will be passed to the finer-level collision detection. For each face $f$ with three corner points $(u, v, w)$ that is adjacent to one of the vertices being passed into the finer level of collision detection, we will calculate the distance between a number of sample points $\alpha u + \beta v + \gamma w$ of the face $f$ with barycentric coordinates $\alpha + \beta + \gamma = 1$ and the current vertex $V$. If at least one of these distances is smaller than the collision threshold, the two corresponding vertices will be marked as merging vertices and will be deactivated. To further speed the performance, a uniform occupancy grid can be superimposed on the domain space for faster collision detection. Each vertex of the object will belong to a grid cell, and each grid cell will store the index/pointer of the vertices that belong to the current grid cell. At the beginning of each deformation step, the occupancy grid needs to update its vertices information. This can be done locally since only a few vertices will move at each deformation step, and it usually takes constant time, or at most $O(n)$.

*Merging-vertices clustering*

After all the merging vertices have been deactivated, we need to divide them into several connected clusters. We randomly pick any merging vertex and find all of its connected merging vertices by a breadth-first search. We recursively do this until all the merging vertices belong to certain merging vertex clusters. Then for each cluster, we will remove all its interior vertices, and put all its boundary vertices into a linked list. We called this algorithm "lazy merging," and is based on the following observation: *when two or more propagating fronts are merging with each other, only the boundary regions will remain, all the interior regions will disappear* (*i.e., removed*).

*Multiple-contours stitching*

After the merging-vertex linked lists have been created, we need to stitch them together. We propose a "multiple-contours stitching" algorithm that is based on the proximity information between merging vertices. The algorithm iteratively connects each vertex in the linked list with its corresponding merging vertex in another linked list, and creates triangle strips that connect the two contours. If there are more than two contours that need to be stitched together, then there may be holes generated in the center of multiple contours. A hole-filling operation is conducted by inserting a new vertex in the center of the gap and connecting it with all the vertices in the gap. Figure 1 illustrates the three steps involved in the "multiple-contours stitching" algorithm.

(1) For each vertex in the linked lists, find its closest vertex in other linked lists.
(2) Based on the proximity information obtained from the previous step, find a pair of vertices $A$ and $B$ such that they are adjacent to each other in the linked list $L$ (see Figure 1(a)), and their closest merging vertices $A'$ and $B'$ are also adjacent to each other in the corresponding linked list $L'$, in addition, the closest merging vertices of $A'$ and $B'$ are $A$ and $B$, respectively. Start from this pair of vertices $A$ and $B$, iteratively go through the linked lists and if possible, connect each pair of adjacent vertices in one linked list to a corresponding vertex in another linked list and create a new triangle. Alternatively, we can search through all pairs of vertices that are adjacent to each other in their respective link lists, and find the closest two pairs of adjacent vertices $A$ and $B$, and $A'$ and $B'$, whose summation $\|A - A'\|^2 + \|B - B'\|^2$ of the square of distance between the corresponding vertices $(A, B)$ and $(A', B')$ is the minimum.
(3) If there are more than two linked lists to be stitched together, then after stitching all the corresponding vertices, there may be some in-between gaps that need to be filled in. For example, in Figure 1(a), there is a gap between the linked lists $L$, $L'$, and $L''$ that consists of vertices $B$, $C$, $C''$, $C'$, and $B'$. We filled in the gap by creating a new vertex $E$ at the center and by connecting the new vertex $E$ with all the other vertices in the loop (see Figure 1(b)).

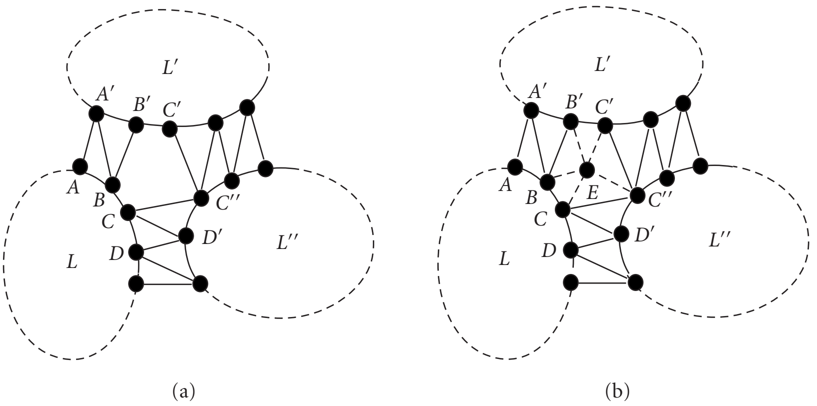

Figure 1: Multiple-contours stitching. (a) New triangles are created by connecting corresponding vertices between different linked lists. A gap that consists of vertices $B$, $C$, $C''$, $C'$, and $B'$ is generated and need to be filled in. (b) The gap is filled in by creating a new vertex $E$ in the center and connecting it with all other vertices in the gap.

*2.4.2. Topology splitting*

Topology splitting occurs when a part of the surface becomes too narrow and intends to shrink itself to a single point. In this scenario, the surface has to split up into two parts precisely at that location. In this paper, we use a method similar to the one proposed by Kass et al. [1]. In particular, if there exist three neighboring vertices which are interconnected to each other, but the face consisting of these three vertices does not belong to the model (i.e., a virtual face), then if the length of any of the three edges of the virtual face is smaller than the minimum edge length threshold and thus needs to be collapsed, a split operation is triggered. For example, in Figure 2, face $ABC$ represents a virtual face that needs to be split because the length of the edge $BC$ is smaller than the threshold. To properly handle the split operation, we divide the surface exactly at this location by cutting it into two open sub-surfaces. Then we close the two split-in-two surfaces using two faces $A1B1C1$ and $A2C2B2$ whose orientations are opposite to each other. Finally, we reorganize the neighborhood around the new faces $A1B1C1$ and $A2B2C2$, while removing the old vertices $A$, $B$, and $C$ from the model.

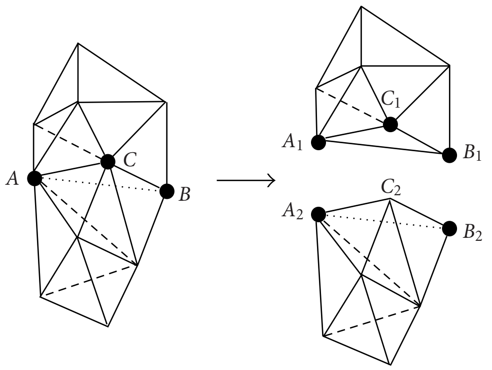

Figure 2: Topology splitting by splitting the virtual face $ABC$ into two faces $A1B1C1$ and $A2C2B2$ whose orientations are opposite to each other.

### 2.5. Model refinement

Once an initial shape of the object is recovered, the model can be further refined several times to improve the fitting accuracy. In this paper, we have implemented two kinds of model refinement: global refinement and local/adaptive refinement. The decision of which method to be employed can be made either interactively by the user (whether he/she prefers a more uniformed mesh or an adaptively sampled mesh), or automatically by the system. The system can make a technically sound decision by calculating the variance of the fitting accuracy of the current model. If the variance of the fitting accuracy is very low, then the underlying object must be relatively smooth and global refinement will be a good choice. Otherwise, adaptive refinement will be used to recover the fine details embedded in the underlying object. Global refinement is conducted by Loop's subdivision scheme [14].

Adaptive refinement is guided by the fitting accuracy. Various kinds of metrics can be used to evaluate the fitting accuracy over each triangle. For example, if the object boundary is well defined, then the fitting accuracy can be defined as the maximum or average distance of the triangle to the object boundary. However, for most of the medical image datasets including those obtained from image modalities such as CT or MRI, the object boundary is unknown; in this case, the curvature of the triangle can serve as the fitting accuracy. The curvature of the triangle can be defined as the summation of the mean curvature of its three vertices, which can be calculated by the aforementioned discrete curvature estimator of (4) in Section 2.2. At each level of adaptive refinement, all the triangles whose fitting accuracy is below the user-specified threshold will be quadrisected. The deformation of the model will resume only among those newly refined regions. Several levels of adaptive refinement can be applied until a user-specified fitting accuracy has been met.

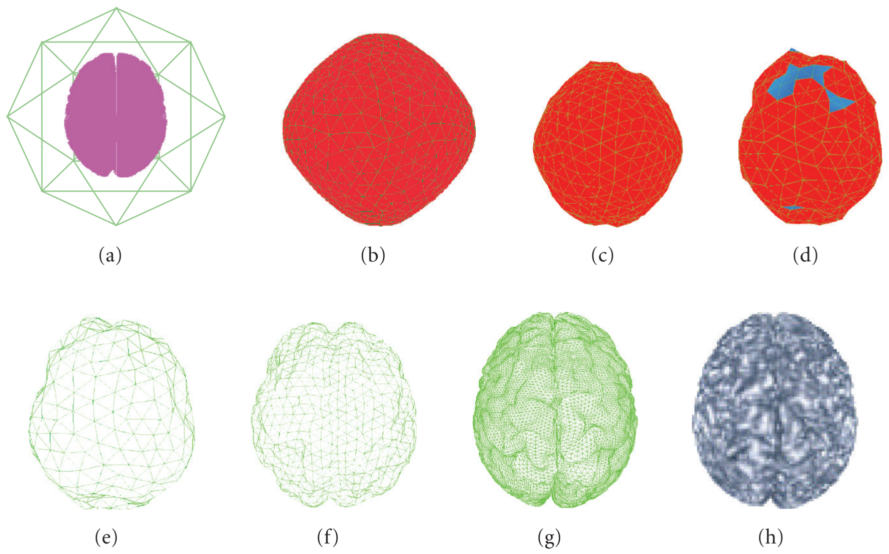

FIGURE 3: Reconstruction and segmentation of an MRI volumetric dataset of a human brain.

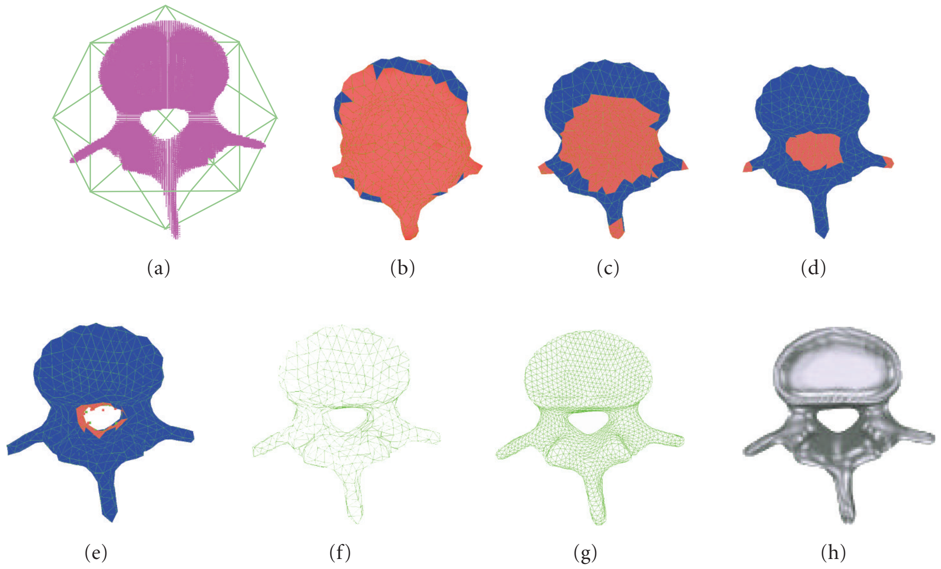

FIGURE 4: Segmentation of a CT volumetric dataset of a human vertebra.

### 3. RESULTS

In this section, we will show some experimental results obtained from our new model on both real and synthetic volumetric datasets in medical imaging applications. Note that in all the following figures, red regions represent parts of the model that are still active and deforming, blue region represent parts of the model that have already reached the boundary of the object and are not active anymore. The input dataset of Figure 3 is an MRI brain dataset of size 91 by 109 by 91 voxels. Figure 3(a) is the seed model (shown as green lines) enclosing the volumetric dataset (shown as cyan points). Figure 3(b) to Figure 3(d) are the three snapshots during the deformation process. Figure 3(e) is the wireframe view of the initial recovered shape. Figure 3(f) is the shape after one level of global refinement. Figure 3(g) is the shape after another level of adaptive refinement. Figure 3(h) is the rendered view of the same model of Figure 3(g). The input dataset of Figure 4 is obtained from CT scanning of a phantom of the vertebra. The data size is 128 by 120 by 52 voxels. Figure 4(a) is the model initialization stage with the seed model enclosing the volumetric dataset. The four figures Figure 4(b) to Figure 4(e) are the snapshots of the model while they are still deforming, we can see that the topology

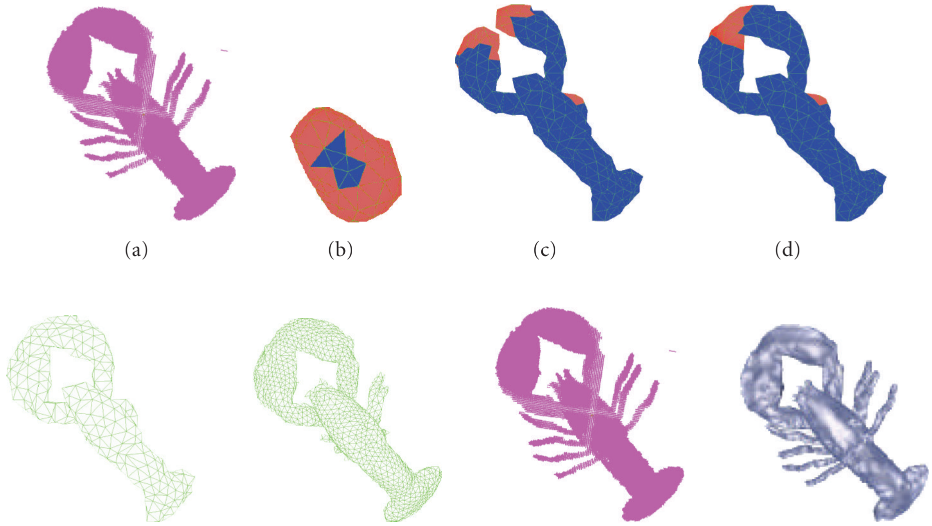

(a) (b) (c) (d)

(e) (f) (g) (h)

Figure 5: Segmentation of a CT volumetric dataset of a lobster.

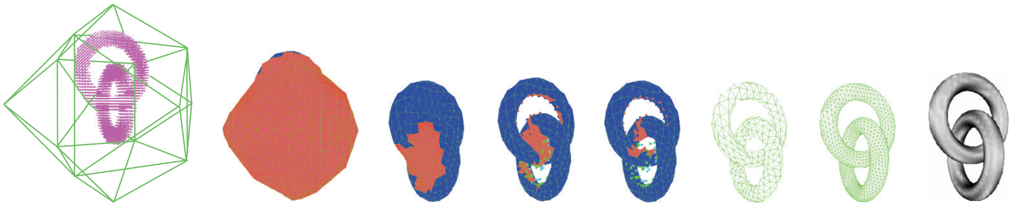

(a) (b) (c) (d) (e) (f) (g) (h)

Figure 6: Segmentation of a synthetic volumetric dataset of the union of two disjoint tori.

of the model has been correctly modified. Figure 4(f) is the wireframe view of initial recovered shape of the model. Figure 4(g) is the refined shape after one level of global refinement. Figure 4(h) is the rendered view of the same shape in Figure 4(g). The input dataset of Figure 5 is a lobster dataset generated by CT scan with 128 by 128 by 128 voxels. Figure 5(a) shows the initial seed model (shown as tiny red dot) inside the volumetric dataset. This illustrates that the new model cannot only shrink from the outside of the dataset, but can also grow from the inside of the dataset. The middle two figures Figure 5(b) and 5(c) are the two snapshots of the model before and after the topology modification. Figure 5(d) is the wireframe view of initially recovered shape. Figure 5(f) is the wireframe view of the shape after one level of global refinement. Figure 5(g) is the shape after another level of adaptive refinement. Highly detailed features such as the tails are well represented. Figure 5(h) is the rendered view of the same model of Figure 5(g). The input of Figure 6 is a synthetic volumetric dataset generated by the union of two disjoint tori with 100 by 100 by 100 voxels. Just like previous examples, Figure 6(a) is the model initialization stage. Figure 6(b) to Figure 6(c) are the two snapshots of the model deformation process. The topology modification process is illustrated in Figure 6(d) to Figure 6(e). Figure 6(f) is the wireframe view of the initial recovered shape. Figure 6(g) is the refined shape after one level of global refinement. Figure 6(h) is the rendered view of the same model in Figure 6(g). Table 1 summarizes the information of the recovered shape, including the number of vertices, edges, and faces for each model, and the running time. The running time is measured on an Intel Pentium 4 M 1.6 GHz Notebook PC with 384 MB internal memory.

### 4. CONCLUSION

We have proposed a topology-adaptive multiresolution deformable surface that is very useful for medical image applications such as boundary reconstruction, surface visualization, data segmentation, and topology discovery. The deformation of the new model is governed by PDEs such as the weighted minimal surface flow. In comparison with existing topology-adaptive explicit models, our model is natural and

Table 1: Running time information.

| Figure no. | Vertices no. | Faces no. | Edges no. | Time (s) |
|---|---|---|---|---|
| 3(e) | 436 | 868 | 1302 | 21 |
| 3(f) | 1666 | 3328 | 4992 | 79 |
| 3(g) | 4762 | 9520 | 16280 | 468 |
| 4(f) | 759 | 1518 | 2277 | 58 |
| 4(g) | 3082 | 6164 | 9246 | 141 |
| 5(e) | 362 | 724 | 1086 | 26 |
| 5(f) | 1693 | 3386 | 5079 | 96 |
| 5(g) | 5591 | 11182 | 16773 | 367 |
| 6(f) | 493 | 986 | 1479 | 28 |
| 6(g) | 1989 | 3978 | 5967 | 23 |

intuitive, easy to use and implement, hierarchically flexible, and has much better control of the model quality and resolution. Significantly different from the level-set approach, our new model always maintains the explicit representation of the geometry and topology, making it a powerful tool for topology interrogation and geometric recovery. More importantly, our model is uniquely suited for modeling time-varying datasets (because of its PDE relevance) such as 3D motion tracking for medical imaging analysis. We are currently exploring research topics along this direction.

### REFERENCES

[1] M. Kass, A. Witkin, and D. Terzopoulos, "Snakes: active contour models," *International Journal of Computer Vision*, vol. 1, no. 4, pp. 321–331, 1988.

[2] L. D. Cohen and I. Cohen, "Finite-element methods for active contour models and balloons for 2-D and 3-D images," *IEEE Transactions on Pattern Analysis and Machine Intelligence*, vol. 15, no. 11, pp. 1131–1147, 1993.

[3] W. E. Lorensen and H. E. Cline, "Marching cubes: a high resolution 3D surface construction algorithm," in *Proceedings of the 14th Annual Conference on Computer Graphicss (SIGGRAPH '87)*, Computer Graphics, pp. 163–169, Anaheim, Calif, USA, July 1987.

[4] D. Terzopoulos, A. Witkin, and M. Kass, "Symmetry-seeking models and 3D object reconstruction," *International Journal of Computer Vision*, vol. 1, no. 3, pp. 211–221, 1987.

[5] R. Malladi, J. A. Sethian, and B. C. Vemuri, "Shape modeling with front propagation: a level set approach," *IEEE Transactions on Pattern Analysis and Machine Intelligence*, vol. 17, no. 2, pp. 158–175, 1995.

[6] V. Caselles, R. Kimmel, G. Sapiro, and C. Sbert, "Minimal surfaces based object segmentation," *IEEE Transactions on Pattern Analysis and Machine Intelligence*, vol. 19, no. 4, pp. 394–398, 1997.

[7] G. Sapiro, *Geometric Partial Differential Equations and Image Analysis*, Cambridge University Press, New York, NY, USA, 2001.

[8] J. A. Sethian, *Level Set Methods and Fast Marching Methods*, Cambridge University Press, Cambridge, UK, 2nd edition, 1999.

[9] T. McInerney and D. Terzopoulos, "Topologically adaptable snakes," in *Proceedings of the 5th International Conference on Computer Vision (ICCV '95)*, pp. 840–845, Cambridge, Mass, USA, June 1995.

[10] T. McInerney and D. Terzopoulos, "Topology adaptive deformable surfaces for medical image volume segmentation," *IEEE Transactions on Medical Imaging*, vol. 18, no. 10, pp. 840–850, 1999.

[11] J.-O. Lachaud and A. Montanvert, "Deformable meshes with automated topology changes for coarse-to-fine three-dimensional surface extraction," *Medical Image Analysis*, vol. 3, no. 2, pp. 187–207, 1999.

[12] Y. Duan, L. Yang, H. Qin, and D. Samaras, "Shape reconstruction from 3D and 2D data using PDE-based deformable surfaces," in *Proceedings of the 8th European Conference on Computer Vision (ECCV '04)*, vol. 3023 of *Lecture Notes in Computer Science*, pp. 238–251, Prague, Czech Republic, May 2004.

[13] Y. Duan, J. Hua, and H. Qin, "Interactive shape modeling using lagrangian surface flow," *The Visual Computer*, vol. 21, no. 5, pp. 279–288, 2005.

[14] C. Loop, "Smooth subdivision surfaces based on triangles," M. S. thesis, Department of Mathematics, University of Utah, Salt Lake City, Utah, USA, August 1987.

[15] M. Desbrun, M. Meyer, P. Schröder, and A. H. Barr, "Implicit fairing of irregular meshes using diffusion and curvature flow," in *Proceedings of the 26th International Conference on Computer Graphics and Interactive Techniques (SIGGRAPH '99)*, Computer Graphics, pp. 317–324, Los Angeles, Calif, USA, August 1999.

[16] L. Kobbelt, T. Bareuther, and H.-P. Seidel, "Multiresolution shape deformations for meshes with dynamic vertex connectivity," in *Proceedings of the 21st Annual Conference of the European Association for Computer Graphics (Eurographics '00)*, pp. 249–260, Interlaken, Switzerland, August 2000.

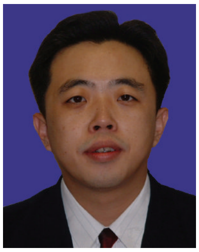

**Ye Duan** is an Assistant Professor of computer science at the University of Missouri-Columbia. He received his Ph.D. degree in computer science from the State University of New York at Stony Brook (2003). He received his M.S. degree in computer science from the State University of New York at Stony Brook in 1998. In 1996, he received his M.S. degree in mathematics from Utah State University. He received his B.A. degree in mathematics from Peking University in 1991. His research interests include biomedical imaging, computer graphics, scientific visualization, computer vision, geometric and physics-based modeling, virtual reality and human-computer interaction, and computer animation and simulation.

